# Assessment of the backscattered skin dose from the beam stopper on Halcyon and Ethos radiotherapy systems

**DOI:** 10.1002/acm2.70299

**Published:** 2025-10-24

**Authors:** Linda Lankinen, Jouko Lehtomäki, Ari Harju, Magdalena Constantin, Taoran Li

**Affiliations:** ^1^ Varian Medical Systems A Siemens Healthineers Company Helsinki Finland; ^2^ Varian Medical Systems A Siemens Healthineers Company Palo Alto California USA

**Keywords:** backscatter, beam stopper, Ethos, Halcyon, Monte Carlo, radiotherapy, skin dose

## Abstract

**Background:**

The backscatter contribution from the beam stopper to the exit‐side skin dose on the Halcyon and Ethos radiotherapy systems has not been studied, and this contribution cannot be separated by experimental methods.

**Purpose:**

This study aimed to assess how the beam backscatter from the beam stopper contributes to the exit‐side skin dose.

**Methods:**

The beam stopper and a simplified model of the bore and MV imager were implemented in a validated Monte Carlo model of Halcyon and Ethos radiotherapy systems. To analyze the worst‐case scenarios of the backscatter, the largest field size was used, and the dose deposition in a water phantom was calculated with four different phantom setups, varying the distance from the bore and the beam stopper. The backscatter contribution to the dose was isolated, and the spectral properties of the backscattered photons were analyzed for one phantom setup.

**Results:**

The depth dose curves and the separated backscatter contribution were presented, with a maximum backscatter contribution of 2% of the local dose at the exit‐side skin dose observed when the phantom was closest to the bore and the beam stopper.

**Conclusions:**

The backscatter contribution to the skin dose was found to be minimal, even with the largest field size and the phantom positioned extremely close to the bore.

## 1 INTRODUCTION

**FIGURE 1:**
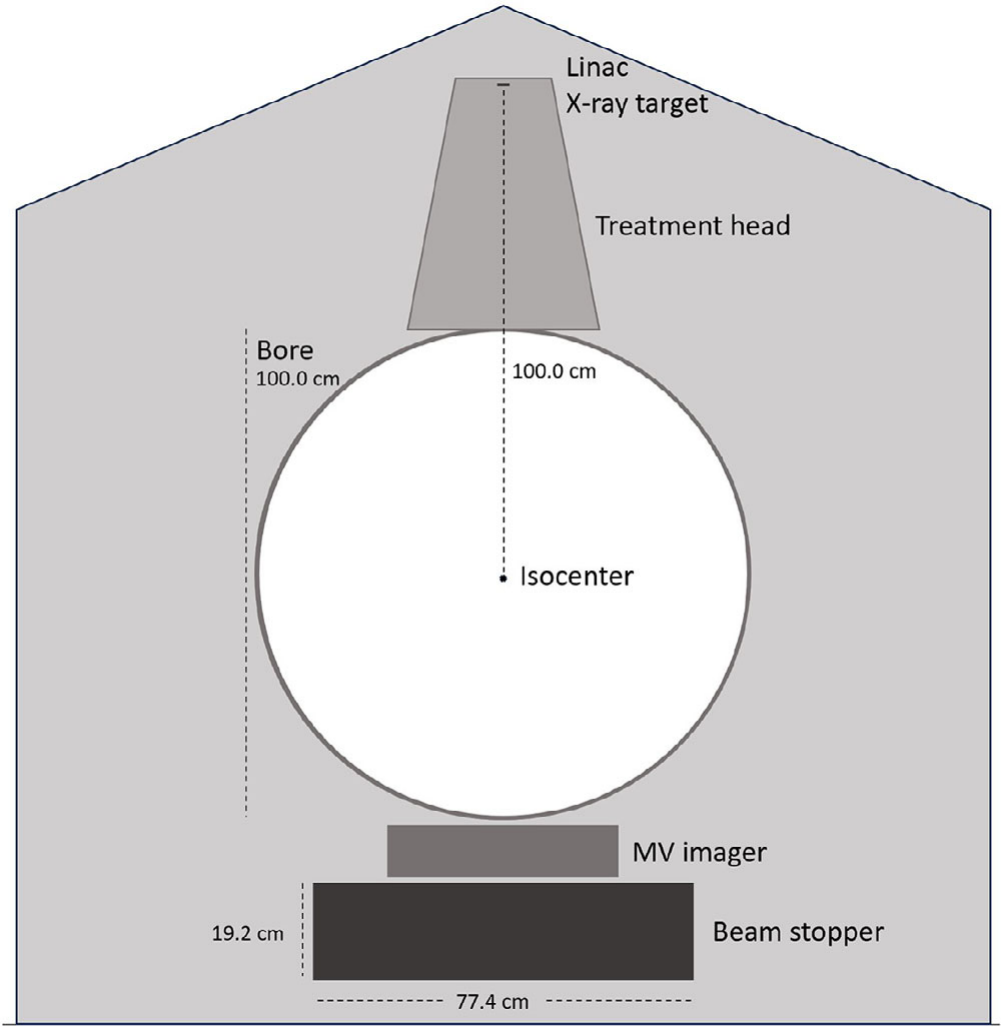
A schematic cross‐sectional view of Halcyon system gantry enclosure, the MV imager, and the beam stopper.

The Halcyon treatment delivery platform is used in the Halcyon and Ethos Radiotherapy systems (Varian Medical Systems, Inc.). The treatment delivery system is designed to provide high‐quality therapy and operational efficiency with minimal installation and operational demands.^1^ The system schematically shown in Figure 1 has an O‐ring design, a single energy 6 MV flattening filter free (FFF) beamline, a treatment head with dual‐layer stacked and staggered multi‐leaf collimator (MLC), and an integrated beam stopper. The integrated MV imaging panel, also known as the electronic portal imaging device (EPID), is placed on top of the beam stopper facing the patient. The linear accelerator (linac) head shield and integrated beam stopper are designed to reduce primary radiation shielding requirements, and the beam stopper also serves as a counterweight to the rotating linac and treatment head.

To underscore the clinical significance, even small variations in superficial dose can be critical in treatments where skin toxicity is a limiting factor, such as in breast or head‐and‐neck radiotherapy. Skin dose, or dose to the superficial region of the patient, is generally sensitive to scattering contributions coming from beamline components. Previous studies have stated that FFF beams have higher entrance skin doses compared to flattening filtered beams due to increased electron contamination.^2^, ^3^ Specifically, in breast treatments with Halcyon's FFF beam, increased superficial skin doses have been reported in comparison with the conventional C‐arm type linacs with flattening filtered beams.^4^, ^5^, ^6^ There have been published studies on the out‐of‐field dose, and the leakage and transmission measurements of the beam stopper in O‐ring design systems,^1^, ^7^, ^8^, ^9^, ^10^ but it is unclear how the exit‐side skin dose of patients is impacted. Additionally, skin dose measurements at the actual depth of the skin target cells (≤ 0.1 mm) are difficult to make, ^3^ and the surface build‐up region is sensitive to the detector type. Detector positioning and readings often need to be corrected and/or extrapolated due to assumptions on the charged particle equilibrium and the perturbation conditions.^11^

Although the backscatter from the beam stopper is expected to be minimal due to its heavy lead alloy construction, the lack of reference data for the exit‐side skin dose raises the possibility that even a small backscatter contribution may be clinically relevant in certain scenarios. If a considerable number of particles undergo backscatter from the beam stopper and reach the patient, they would contribute to the skin dose. However, such a contribution has not been quantified. Moreover, the MV imager or EPID is placed directly on the beam stopper, and its backscatter effect has not been investigated. Lastly, the Halcyon treatment delivery system also has a bore cover, which could reduce the backscatter contribution to skin dose. To understand in detail how much distal skin dose is contributed from backscatter, this study simulated and analyzed the backscattered radiation from the beam stopper and other components (i.e., EPID) on Halcyon treatment delivery platform using a Monte Carlo (MC) model ^12^, ^13^.

## 2 METHODS

### 2.1 MC model implementation

The beam stopper and a simplified model of the bore and MV imager were implemented into the validated MC model of the Halcyon and Ethos radiotherapy system.^12^ The MC model acts as a computational replica of an actual machine, providing reference data beyond the capabilities of experimental methods, as there is no practical way to separate the backscatter particles from the direct radiotherapy beam. The bore envelope was set as a cylinder 50 cm from the isocenter. The system is equipped with an amorphous silicon (a‐Si) photodiode‐based EPID (aS1200, Varian Medical Systems, Palo Alto, CA), which is designed for the daily check of the machine performance and as tool for plan‐specific pre‐treatment QA. The MV imager was modeled with simplified geometries representing the materials and thicknesses of the scintillator and the supporting layers. The imager is a flat‐panel system mounted permanently inside O‐ring gantry at a fixed source‐to‐imager distance (SID) of 154 cm. The active area covers 43 cm × 43 cm and a 1280 × 1280 pixel matrix, translating into an imaging area of 28 cm × 28 cm.^14^ EPID consists of a buildup copper plate ( 1mm, 8.9 ${\text{g/cm}}^{3}$), a phosphor scintillator (0.49 mm), an amorphous silicon flat panel array detector (0.7 mm, 2.4 ${\text{g/cm}}^{3}$), an aluminum plate (1 mm, 2.7 ${\text{g/cm}}^{3}$), and a lead plate (3 mm, 11 ${\text{g/cm}}^{3}$) for backscatter shielding ^15^.

The beam stopper was installed beneath the digital megavolt (MV) imager panel, as shown in Figure 1. The beam stopper dimensions are 75.4 cm × 66 cm × 17.2 cm in *X* (couch lateral direction left‐right), *Y* (couch longitudinal direction in‐out), and *Z* (couch vertical direction up‐down) directions, defined following the standard IEC 61217 conventions.^1^ The beam stopper is constructed as lead alloy with 3% antimony encased in a 10 mm thick steel box placed at 62.6 cm from the isocenter.^8^

### 2.2 Phantom setups

**FIGURE 2:**
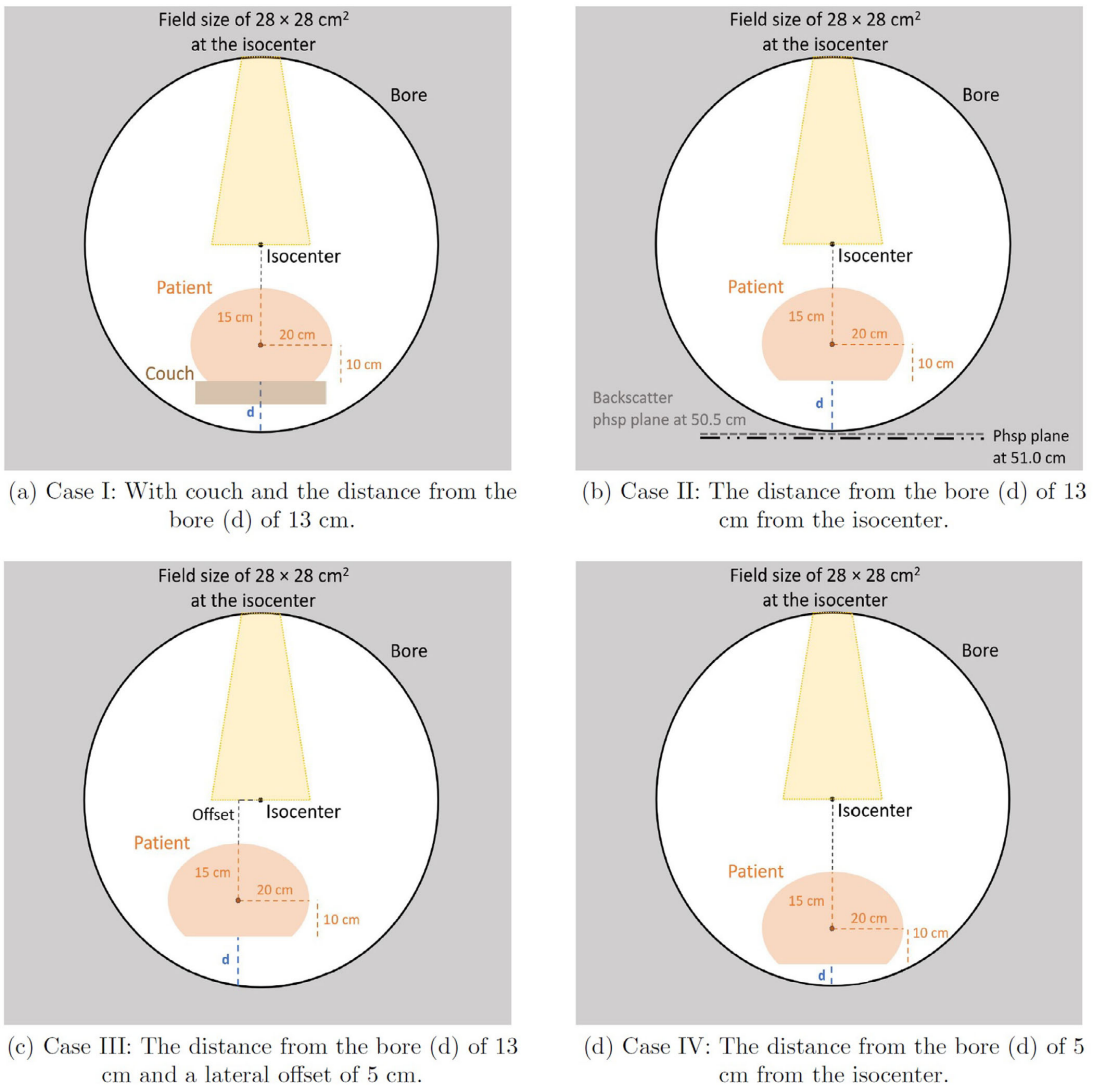
A schematic illustration of the size and the locations of the partial elliptical cylinder phantom inside the bore and the distance from the bore (d). Case II setup (b) also shows the locations of the first (black long dash dotted line) and the backscatter (gray dashed line) PHSP planes at 51.0 cm and 50.5 cm downstream from the isocenter.

A water phantom was used with four different setups (Cases I–IV) presented in Figure 2a–d. The phantom was a 30 cm long partial elliptical cylinder with main diameters of 40 and 25 cm. The phantom was voxelized to allow scoring the percent depth dose and the skin dose on the distal end of the water phantom. The offsets from the bore (d) varied. The set of test cases was selected to describe the worst‐case scenarios of patient setup for the simulation of the backscatter radiation contribution to skin dose. Case I was representative for the treatments where the patient has an offset closer to one side of the bore, and the beam travels through the patient and the couch with zero‐degree gantry. The couch was removed from the cases (II‐IV) to estimate the scenarios where the beam is not attenuated by the couch. Otherwise, the patient setup in Case II was the same as in Case I. Case III had an additional lateral offset of 5 cm so that the direct beam aperture extended beyond the patient, increasing the portion of unattenuated beam directed towards the beam stopper. Case IV was representative of treatment plans where the patient is extremely close to the bore. However, the system will alert if the distance between the patient geometry and bore is less than 5 cm.

### 2.3 MC simulations

The Geant4 physics list “QGSP_BIC” with “EMOpt4” was used in the simulations.^12^ The production cuts for secondary particle generation were set to 0.1 mm, resulting in energy thresholds in water of 1.11 keV for photons, 84.66 keV for electrons, and 83.53 keV for positrons. Secondary particles below these thresholds were not tracked, and their energies were locally deposited. The simulations were divided into two stages to separate the backscatter contribution to the skin dose.

#### 2.3.1 First stage simulations

The particle transport was simulated starting with initial field‐independent phase space (PHSP) files between the secondary collimator and MLC at a distance of 69.4 cm from the isocenter.^12^ A total of 5 billion incident electrons were used. The largest field size with fully open MLC (28 cm × 28 cm) for 0‐degree collimator rotation was used to maximize the production of the backscattered particles with unattenuated beam hitting the beam stopper. The dose deposition in the phantom was calculated. A PHSP plane at ‐51 cm downstream from isocenter capturing all particles traveling towards the MV imager and the beam stopper was recorded (long dash dotted line) in Figure 2b). All particles transported to the PHSP plane were stopped and captured.

#### 2.3.2 Second stage simulations

The recorded PHSP plane at ‐51 cm downstream from isocenter was used as an input, and the particles were transported to the MV imager and the beam stopper. To isolate the backscatter contribution, a second PHSP plane at ‐50.5 cm downstream from the isocenter was recorded (gray dashed line in Figure 2b). The second PHSP captured only the backscattered particles traveling back towards the patient, as it was located upstream from the first stage PHSP and closer to the isocenter. The dose deposition from the backscatter radiation in the phantom was calculated, and the contribution to the skin dose was determined.

### 2.4 Data analysis

The dose deposition from the first stage simulations was compared with the backscattered dose deposition. The depth dose curves from the surface to the distal end of the phantom (0 to 250 mm) were compared along the central axis of the phantom, that is the line through the center of the elliptical phantom. A resolution of 2 mm was used in the depth direction and 24 mm in a plane perpendicular to the depth direction. At the distal end of the phantom, the resolution in the depth direction was increased to 0.02 mm on the last voxel from 248 to 250 mm. The statistics of the dose deposit in the second stage simulations were increased by recycling the incident PHSP files three times.

The energy spectrum of the backscattered photons at ‐50.5 cm downstream from the isocenter was analyzed for Case II setup, as this case represented beams which traveled through the patient with varying attenuation across the field. The spectrum of the backscattered photons was compared to the energy spectrum of all photons at 51.0 cm downstream from the isocenter. The spectra were normalized by the total number of simulated particles and the energy bin width to obtain the energy spectra. The angular dependence of the backscatter photons at ‐50.5 cm downstream from the isocenter was studied by dividing the plane into rings representing different angular divergences from the beam axis.

## 3 RESULTS

### 3.1 Depth dose curves and the backscatter contribution

**FIGURE 3:**
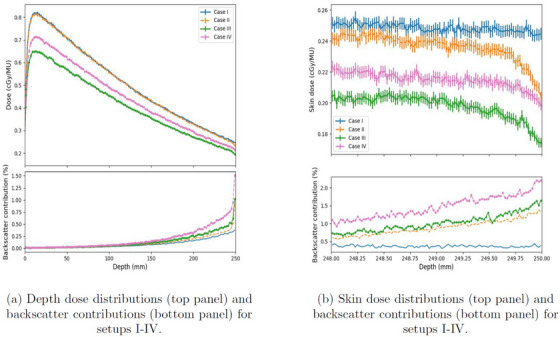
Depth dose (a) and skin dose distributions (b) for setups I–IV.

Depth dose curves and the backscatter dose contribution along the central line of the phantom with the different phantom setups (I–IV) are presented in Figure 3a. The total depth dose curves, including the backscatter contribution, are shown on the top, and the backscatter contributions are shown on the bottom. The surface of the phantom was at 0 mm, and the distal end of the phantom was at 250 mm. The contribution of the backscatter dose was below 2% in all cases (I–IV). Cases I and II had the highest depth dose but lowest backscatter contribution as the distance from the beam stopper was 13 cm. Case I with the couch had a smaller increase in the backscatter contribution at the distal end of the phantom than Case I without the couch. Case III with lateral offset had the lowest depth dose with a small increase in the backscatter contribution at the distal end of the phantom. Case IV had lower depth dose compared to Cases I and II, but the distal end of the phantom had an increased backscatter contribution of 1.5%. However, the total skin dose at the distal end was smaller than in Cases I and II. Case I had the smallest backscatter contribution. When removing the couch in Case II, the total skin dose was nearly unchanged, but the backscatter contribution increased compared to Case I from 0.4% to 0.9%. In Case III, the total skin dose dropped, but the ratio of backscatter to total skin dose increased to 1.0%. By bringing the phantom extremely close to the beam stopper and decreasing the distance to 5 cm from the bore, the ratio increased to 1.5%, although the total skin dose was smaller than in Cases I and II.

In Figure 3b, the total skin dose and the backscatter contribution in a 2 mm region at the distal end of the phantom are shown with higher resolution. Case I shows a constant skin dose and backscatter contribution. Cases II to IV had a decreased dose at the distal end of the phantom with increased backscatter contribution. The largest backscatter contribution of 2 % was observed at the distal end of the phantom in Case IV. In the Supplementary Material (SM), the field size dependency is presented using smaller field sizes: 4 cm × 4 cm, 10 cm × 10 cm, and 15 cm × 15 cm.

### 3.2 Energy spectra of the backscattered photons

**FIGURE 4:**
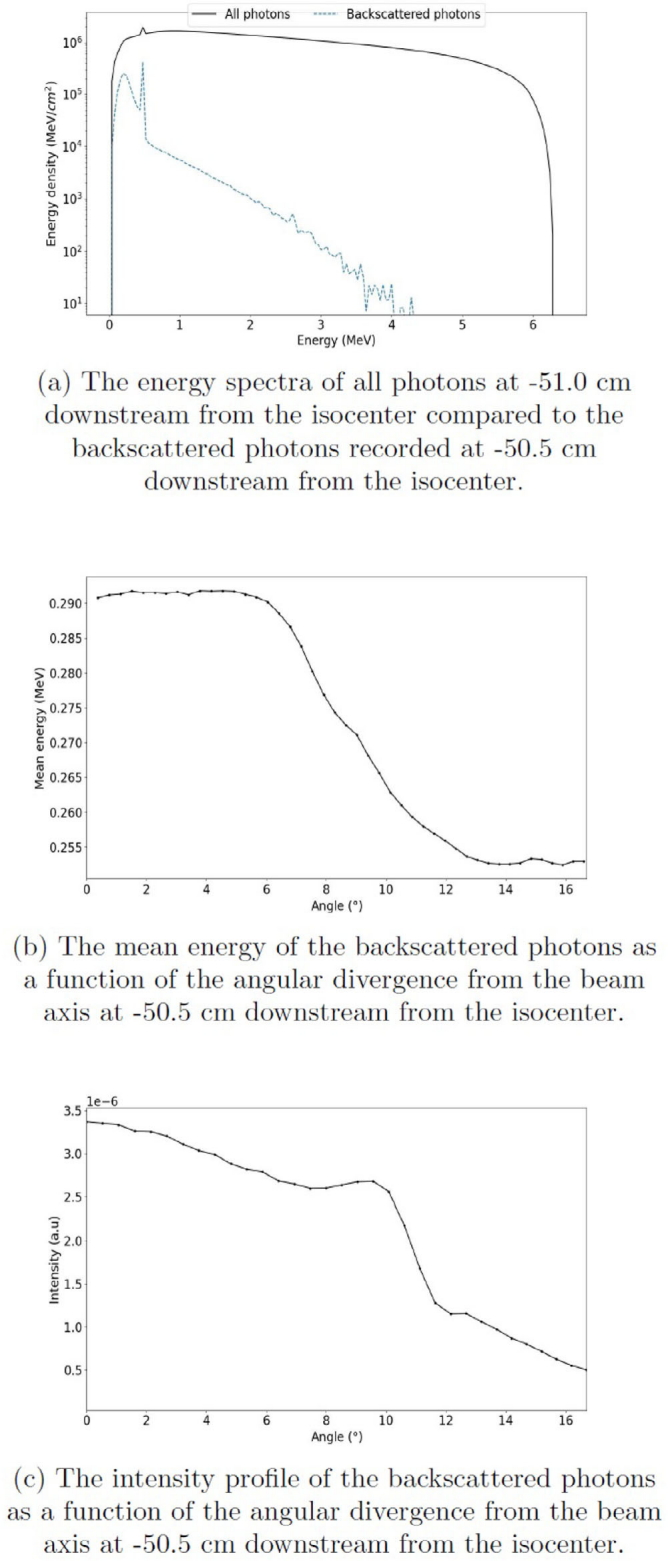
The energy spectra, the mean energy, and intensity profile of the backscattered photons collimated with an open beam of 28 cm × 28 cm and Case II phantom setup.

To characterize the energy spectra, the largest field size of 28 cm × 28 cm and Case II setup were used, and the photons were recorded in PHSP planes above the MV imager. In Figure 4a, the energy spectra of all photons at ‐51.0 cm downstream from the isocenter (black solid line) and the backscattered photons at ‐50.5 cm downstream from the isocenter (blue dashed line) are presented on a logarithmic scale. The spectrum for all photons has more constant distribution of energies up to 6.3 MeV with a mean energy of 1.2 MeV. In comparison, the spectrum of the backscattered photons shows energies mostly below 2 MeV with a mean energy of 0.27 MeV. Both spectra show a peak at 511 keV originating from annihilation photons. The spectrum of the backscattered photons shows a combination of the characteristic peaks for lead between 72–88 keV (K‐shell edge) and 9‐13 keV (L‐shell edge). The ratio between the number of backscattered photons and the total number of all photons was 6.9 %.

The mean energy of the backscattered photons as a function of the angular divergence from the main beam axis is shown in Figure 4b. The mean energy was constant at 0.29 MeV up to 6 degrees and then decreased to 0.25 MeV between 6 and 13 degrees. The intensity profile of the backscattered photons is presented in Figure 4c. The profile shows a peak at the centerline and a small increase in intensity from 8 to 10 degrees. The intensity increased as the beam was less attenuated by the phantom on the edge of the field.

## 4 DISCUSSION

In this study, the backscattered skin dose was found to have a small contribution to the total skin dose near beam exit, that is, below 2% of exit skin dose for all investigated testing scenarios. The maximal dose from backscatter in the worst‐case scenario is around 0.01–0.04 mGy per MU (0.1%–0.4% of primary dose at reference condition). This contribution potentially has the largest impact for large fields, 2D or 3D treatment. For example, for a typical opposing beam 2D treatment with 200 cGy per fraction prescribed at midline involving around 200 MU per field, the backscatter contribution to skin dose will be around 2–8 mGy, which puts it on a similar magnitude as kV CBCT imaging dose from a treatment fraction.^16^ For more complex treatment with higher MUs and more spread‐out beam exiting area on the skin (e.g., IMRT and VMAT), the contribution from backscatter will likely be similar to dose from linac head leakage (0.1% of primary beam).

The attenuating effect of the couch on backscattered photons was found to be minimal but not negligible. The presence of the couch was found to attenuate the backscattered photons slightly; cases without the couch exhibited a modestly higher relative backscatter component, particularly for lateral offsets. Moreover, as the patient (or phantom) is positioned closer to the bore or beam stopper, the relative backscatter contribution increases, although the total skin dose decreases due to decreased fluence at a larger distance from the source.

The spectral characteristics and contribution to skin dose from backscattered photons from high‐Z materials downstream of exiting beam were investigated with open beam and the largest field size. The spectrum of the backscattered particles predominantly exhibited low energies up to 2 MeV, compared to the full open beam spectrum, which reached energies up to approximately 6 MeV. Inside the field, the mean energy of the backscattered photons was constant at 0.29 MeV with respect to the angle. An increased intensity was observed in the center of field, as expected, and in the corners of the field due to the less attenuation in the phantom geometry at the field edges.

The limitations of this study include the simplified phantom geometries in the MC model, which may not fully capture the complexity of patient anatomy or the full range of beam interactions in a clinical setting. Future work is underway to incorporate anthropomorphic phantoms and in‐vivo measurements to validate these simulation results. Heterogeneous tissues may introduce particularly strong perturbations in the dose distributions with low‐density materials, such as lung tissues, and for high‐density materials, such as bone, which represent the extremes of the human tissue density range. The backscatter dose contribution was found to be largest for the skin dose and decreasing to under 0.25% within 5 cm in all cases, and the dose in water would be lower than in low‐density materials and higher than in high‐density materials. Additionally, investigating the dynamic aspects of treatment delivery (e.g., varying gantry angles and using actual patient CT) could further demonstrate the clinical relevance of backscatter contributions for real clinical scenarios.

Despite the limitations, no alarming trends were found even in the worst‐case scenarios: the largest field size and closest distance between patient and beam stopper. This serves as a reassurance for the most impacted clinical treatment cases, such as the breast treatments with oblique gantry angles and the patient shifted laterally closer to the bore.

## 5 SUMMARY AND CONCLUSIONS

The backscatter radiation contribution to exit‐side skin dose from the beam stopper was studied using a Monte Carlo model for the Halcyon treatment delivery platform. The results indicated that the backscatter from the beam stopper in the Halcyon radiotherapy system contributes marginally to the total skin dose, and is comparable to a kV CBCT acquisition. For a 200 cGy fraction delivered with 200 MU, the additional skin dose is ≤ 8 mGy (≤ 4% of prescription).

## AUTHOR CONTRIBUTIONS

All authors contributed to the design and implementation of the research, the analysis of the results, and writing the manuscript. Linda Lankinen performed data collection and data analysis.

## CONFLICT OF INTEREST STATEMENT

All authors are employees of Varian Medical Systems, Inc.

## Supporting information



Supporting Information

## References

[1] Cai B , Laugeman E , Hsu H , et al. Technical note: self‐shielding evaluation and radiation leakage measurement of a jawless ring gantry linac with a beam stopper. Med Phys. 2021;48:3143‐3150. 33763897 10.1002/mp.14858

[2] Mohammed M , Chakir E , Boukhal H , Mroan S , and El Bardouni T . Evaluation of the dosimetric characteristics of 6MV flattened and unflattened photon beam. J King Saud Univ Sci. 2017;29:371‐379.

[3] Xiao Y , Kry FS , Popple R , et al. Flattening filter‐free accelerators: a report from the AAPM therapy emerging technology assessment work group. J Appl Clin Med Phys. 2015;16:12‐29. 10.1120/jacmp.v16i3.5219 PMC5690108 26103482

[4] O'Grady F , Barsky RA , Anamalayil S , et al. Increase in superficial dose in whole‐breast irradiation with Halcyon straight‐through linac compared with traditional C‐arm linac with flattening filter: in vivo dosimetry and planning study. Adv Radiat Oncol. 2020;5:120‐126. 32051898 10.1016/j.adro.2019.07.011 PMC7004930

[5] Seok HJ , Ahn HS , Ahn SW , et al. Comparison of skin dose in IMRT and VMAT with TrueBeam and Halcyon linear accelerator for whole breast irradiation. Phys Eng Sci Med. 2024;47:443‐451. 38224383 10.1007/s13246-023-01373-x PMC11166860

[6] Demir H , Gul VO , Aksu T . Investigation of skin dose of post‐mastectomy radiation therapy for the Halcyon and tomotherapy treatment machine: comparison of calculation and in vivo measurements. Radiat Meas. 2024;173:107112.

[7] Caravani K , Murry R , Healy B . Characterisation of in‐room leakage and scattered radiation for the Varian Halcyon linear accelerator. Phys Eng Sci Med. 2022;45:73‐81. 34797532 10.1007/s13246-021-01084-1

[8] Kaur A , Sahani G , Shrivastava A , and Pawaskar NP . Optimization of radiation shielding considerations for designing Halcyon vault. J Med Phys. 2023;48:1‐12. 37342599 10.4103/jmp.jmp_86_22 PMC10277303

[9] Ramsey RC , Seibert R , Mahan LS , Desai D , and Chase D . Out‐of‐field dosimetry measurements for a helical tomotherapy system. J Appl Clin Med Phys. 2006;7:1‐11. 10.1120/jacmp.v7i3.2212 PMC5722430 17533339

[10] Balog J , Lucas D , DeSouza C , Crilly R . Helical tomotherapy radiation leakage and shielding considerations. Med Phys. 2005;32:710‐719. 15839342 10.1118/1.1861521

[11] Apipunyasopon L , Srisatit S , Phaisangittisakul N . An investigation of the depth dose in the build‐up region, and surface dose for a 6‐MV therapeutic photon beam: Monte Carlo simulation and measurements. J Radiat Res. 2013;54:374‐382. 23104898 10.1093/jrr/rrs097 PMC3589935

[12] Laakkonen L , Lehtomäki J , Cahill A , Constantin M , Kulmala A , and Harju A . Monte Carlo modeling of Halcyon and Ethos radiotherapy beam using CAD geometry: validation and IAEA‐compliant phase space. Phys Med Biol. 2023;68:044002. 10.1088/1361-6560/acb4d9 36657172

[13] Lankinen L , Kulmala A , Lehtomäki J , and Harju A . The delivery assessment for small targets on Halcyon radiotherapy system – measured and calculated dose comparison. J Appl Clin Med Phys. 2024;25:e14407. 38775807 10.1002/acm2.14407 PMC11163489

[14] Razinskas G , Schindhelm R , Sauer AO , and Wegener S . Sensitivity and Specificity of Varian Halcyon's portal dosimetry for plan‐specific pre‐treatment QA. J Appl Clin Med Phys. 2023;24:e14001. 37086428 10.1002/acm2.14001 PMC10402680

[15] Shi M , Myronakis M , Hu Y‐H , et al. A novel method for fast image simulation of flat panel detectors. Phys Med Biol. 2019;64:095019. 30901759 10.1088/1361-6560/ab12aa PMC12290521

[16] Ding GX , Munro P . Radiation exposure to patients from image guidance procedures and techniques to reduce the imaging dose. Radiother Oncol. 2013;108:91‐98. 23830468 10.1016/j.radonc.2013.05.034

