# Supplementary material for: Assessment of the backscattered skin dose from the beam stopper on Halcyon and Ethos radiotherapy systems
Source: J Appl Clin Med Phys. 2025 Oct 24;26(11):e70299. doi: 10.1002/acm2.70299 (PMC12551592; doi:10.1002/acm2.70299)
Supplement: Supplementary file 1 — Supporting Information [file ACM2-26-e70299-s001.pdf]

Supplementary Material for “Assessment of the backscattered skin dose from the beam stopper on Halcyon and Ethos Radiotherapy Systems”

Supplementary Note 1

To study smaller field sizes, Case IV was used as the backscatter contribution was largest and the phantom was closest to the bore and the beam stopper. In Figure S1, the depth dose distributions, the skin doses, the backscatter contributions with different field sizes for Case IV are presented. The dose and backscatter contribution decreased as the field size decreased. The threshold where the backscatter contribution would fall below 1% of exit skin dose would be for the field sizes below 10 cm x 10 cm. The backscatter contribution on the surface dropped from 2% for the largest field size to 1.5% for 15 cm x 15 cm, 1.2% for 10 cm x 10 cm, and 0.25% for 4 cm x 4 cm.

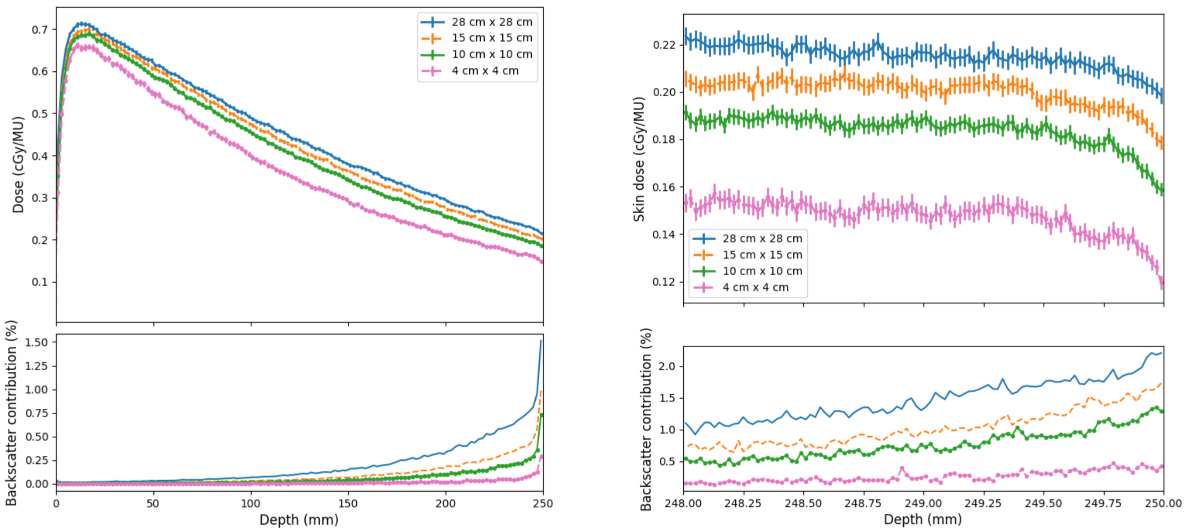

Figure S1 Depth dose, skin dose, and backscatter contribution distributions with different field sizes 4 cm x 4 cm, 10 cm x 10 cm, 15 cm x 15 cm, and 28 cm x 28 cm.
